# Breakthrough Pneumocystis jirovecii Pneumonia in an Allogeneic Hematopoietic Stem Cell Transplant Recipient

**DOI:** 10.7759/cureus.61890

**Published:** 2024-06-07

**Authors:** Tarek Zieneldien, Janice Kim, John Greene

**Affiliations:** 1 College of Arts and Sciences, University of South Florida, Tampa, USA; 2 Department of Internal Medicine, Moffitt Cancer Center, Tampa, USA

**Keywords:** pneumocystis jirovecii, acute myeloid leukemia, graft-versus-host disease, allogeneic hematopoietic stem cell transplant, pneumocystic pneumonia

## Abstract

*Pneumocystis carinii *pneumonia (PCP), which is currently referred to as *Pneumocystis jirovecii* pneumonia, is an opportunistic fungal infection that commonly affects immunocompromised patients, and it is potentially fatal. Individuals at risk include those whose host immunity has been altered by underlying disease states, such as HIV and cancer patients, as well as transplant recipients and those taking immunosuppressive medications. Here, we present a case of a breakthrough PCP infection of an adult allogeneic hematopoietic stem cell transplant patient who was infected despite prophylaxis with inhaled pentamidine. The patient’s transplant course was complicated by acute graft-versus-host disease (GVHD), which was treated with tacrolimus, prednisone, beclomethasone, and budesonide. Treatments for GVHD, which include immunosuppressive therapies, are a risk factor for PCP. Thus, the patient was on prophylactic treatment with inhaled pentamidine. The case presents challenges that immunocompromised patients face, particularly those undergoing allogeneic hematopoietic stem cell transplantation. While the patient received prophylactic treatment, there was still a breakthrough PCP infection. We highlight the risks this infection can cause and the need to promptly address these infections to prevent complications and optimize prophylactic regimens.

## Introduction

*Pneumocystis carinii* pneumonia (PCP), which is now recognized as *Pneumocystis jirovecii* pneumonia, is an opportunistic fungal infection caused by *P. jirovecii* [1]. PCP is a major cause of illness and death in individuals with impaired immune systems. Usually, patients at risk include those taking immunosuppressive medications, as well as those with underlying diseases that alter host immunity, such as human immunodeficiency virus (HIV), cancer, and transplant recipients [2]. Between 1980 and 2000, most PCP cases involved individuals with AIDS. Despite this, PCP has become more prevalent among non-HIV-infected patients because of improvements in the treatment of AIDS as well as more individuals receiving immunosuppressive therapy [3]. Nonetheless, AIDS patients have an overall mortality rate of approximately 10-20%, while non-HIV-infected patients have an overall mortality rate of 35-55% [4]. Patients with PCP exhibit symptoms such as dry cough, low-grade fever, hypoxemia, tachypnoea, and dyspnea [2].

In humans, *P. jirovecii* resides predominantly in the pulmonary alveoli. Morphological research has elucidated three different stages: the trophozoite, or trophic form, which typically is found in clusters, the sporozoite, or precystic form, and the cyst, which possesses various intracystic bodies and is much larger than the trophic form [5]. The cyst wall of *Pneumocystis *is composed of the complex branching polysaccharide β-glucan [6]. There are studies suggesting that interactions of lung cells with the cyst wall can activate innate immune responses [6]. Although microscopic analysis of respiratory specimens remains the standard for diagnosis, polymerase chain reaction (PCR) has been highly effective. PCR has demonstrated the ability to detect DNA of the pathogen in sputum with high sensitivity. Even more, serum β-D-glucan is also an effective adjunctive diagnostic method [7]. Consequently, high-resolution CT is another crucial tool that can show diffuse ground-glass opacities, which thereby yields valuable insight for the assessment of immunocompromised individuals with suspected PCP when chest radiography yields normal results [8]. As of now, treatments against *Pneumocystis *pneumonia mainly rely on trimethoprim-sulfamethoxazole (TMP-SMX) or pentamidine, with increasing concerns about the potential of drug resistance emerging because of widespread use of therapeutics such as TMP-SMX, which is the most common treatment [9]. However, if the patient experiences side effects, intolerance, or resistance to TMP-SMX, there are also alternate treatments including clindamycin and primaquine that have shown to be effective alternative therapies [10].

We present the case of an adult allogeneic hematopoietic stem cell transplant patient who demonstrated a breakthrough PCP infection while obtaining inhaled pentamidine. For this patient, her transplant course was further complicated by severe acute graft-versus-host disease (GVHD). As it is a potentially fatal infectious complication, international guidelines recommend prophylaxis for six months or longer following allogeneic hematopoietic stem cell transplantation (allo-HSCT). Individuals with GVHD or on immunosuppressive therapy are recommended to undergo even longer prophylaxis [11].

## Case presentation

A 50-year-old female, with a hematological history of myelodysplastic syndrome with multilineage dysplasia (MDS-MLD) that has progressed to acute myeloid leukemia (AML), presented with a breakthrough PCP infection on February 14, 2024. The PCP infection was confirmed by a positive PCP stain on bronchoalveolar lavage (BAL), despite prophylaxis with inhaled pentamidine, which is a preventative measure. The patient was initially diagnosed with MDS-MLD in April of 2022. After discussing treatment options, the patient was started on MCC 20716 (azacitidine plus magrolimab/placebo) trial. The patient completed a total of three cycles and was bridged to an allo-HSCT with a matched unrelated donor (MUD) in September of 2022. This procedure was complicated by severe acute GVHD for which she continues tacrolimus and prednisone, which has led to steroid-induced hyperglycemia and hypertension, as well as beclomethasone and budesonide. After the transplant, the patient experienced disease progression and began treatment with decitabine and cedazuridine in September of 2022, which was complicated by respiratory failure and inadequate recovery of blood cell counts, prompting a hold on further cycles. Since the relapse of her disease, she completed two cycles of decitabine and had a bone marrow biopsy conducted on June 12, 2023, demonstrating disease progression to AML. The patient was admitted and then treated with cytarabine and venetoclax, which was started on June 26, 2023. The patient underwent a bone marrow biopsy that showed no evidence of AML, and next-generation sequencing indicated clearance of TP53. Nonetheless, because of incomplete count recovery and complications with cytomegalovirus (CMV) retinitis, she has been unable to proceed with further treatment. When the patient was diagnosed with CMV retinitis, she had an intravitreal foscarnet procedure and was given maribavir and weekly intravitreal injections. After she completed her course of maribavir, she continued letermovir as CMV prophylaxis permanently. The patient experienced diarrhea and was found to have GVHD and was placed on pentamidine prophylaxis.

**Figure 1:**
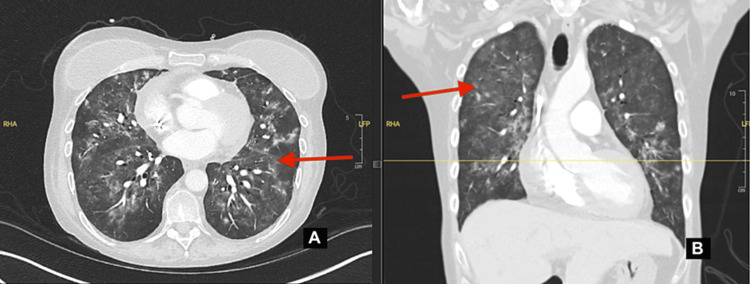
Axial (A) and coronal (B) CT pulmonary angiographies demonstrating extensive bilateral ground-glass opacities and septal thickening.

In February 2024, the patient complained of worsening hypoxia and shortness of breath for one week. She stated that she had needed to use home oxygen therapy of up to five liters via nasal cannula given SpO_2_ around the mid-80s on exertion. Home oxygen was prescribed last May after treatment for cryptogenic organizing pneumonia and possible transfusion-related acute lung injury. The patient stated that she was recently at a bone marrow transplant clinic where a respiratory viral panel was done and was positive for rhinovirus. A chest X-ray was ordered, but it did not reveal any acute findings. She reported a temperature of 100.6 °F at home and intermittent low-grade temperatures and chills. The patient was admitted from February 14, 2024, to February 26, 2024, where she was diagnosed with PCP and broke through the pentamidine prophylaxis. The patient's CT pulmonary angiography showed septal thickening and bilateral ground-glass opacities (Figure 1). During the hospital stay, the patient received supplemental oxygen at a flow rate of two liters per minute. The patient was started on TMP-SMX and prednisone for PCP; however, because of acute kidney injury and transaminitis, the TMP-SMX was changed to clindamycin and primaquine with an end date of March 7, 2024. Nine days were spent in the progressive care unit because of acute respiratory failure, and the patient required a high-flow nasal cannula to assist with breathing. The patient was discharged on February 26, 2024, with O_2_ for 6 L with ambulation and 2 L at rest. On March 3, 2024, the patient returned for neutropenic fevers of 101.3 °F with negative blood cultures but with concerns of pneumonia. The patient was started on empirical antibiotic therapy with ceftriaxone and azithromycin to treat atypical pneumonia, which was completed on March 8, 2024. As of April 25, 2024, the patient has been doing better and feeling less tired, off oxygen but continuing to take atovaquone prophylaxis.

These findings are indicative of the patient’s breakthrough PCP infection, providing different perspectives on the bilateral pulmonary involvement.

## Discussion

Among non-HIV cases, individuals with hematological malignancies, as well as those with recent stem cell or organ transplants and who are on immunosuppressants, have an increased risk of developing PCP infection. Moreover, PCP tends to have a higher fatality in non-AIDS patients, with time to diagnosis affecting survival and often being longer for non-AIDS patients [12]. *P. jirovecii *can be a fatal disease leading to death because of sudden respiratory failure if not recognized early in the course of illness as patients will undergo multiple antibiotic regimens to treat broad symptoms before they are diagnosed and receive proper treatment for the infection [13]. In this case report, we illustrate some of the common risk factors for PCP infections including respiratory failure, inadequate recovery of blood cell counts, steroid use, and hypoxia. To our knowledge, successful and durable treatment of PCP infections responds best to prophylactic TMP-SMX and is the preferred preventative application to reduce the incidence of PCP in HIV-positive immunocompromised patients [14]. As such, TMP-SMX has been seen to significantly reduce PCP infections in HIV-negative immunocompromised patients, resulting in lower mortality rates [15].

As for allogeneic hematopoietic cell transplantation recipients, PCP is a potentially life-threatening complication [16]. Nonetheless, limited research has specifically focused on the epidemiology of the disease within the allogeneic hematopoietic cell transplantation population. There have also been case-control studies demonstrating that PCP develops later posttransplantation than previously reported, supporting extending prophylaxis beyond six months for many patients [16]. Furthermore, there are also alternative medications including pentamidine, dapsone, and atovaquone. Pentamidine for PCP prophylaxis can be administered via inhalation or intravenously. It is typically utilized when patients cannot tolerate TMP-SMX, which is usually the first line of defense for PCP infections, with intravenous administration having shown a 0.7% breakthrough rate in a meta-analysis [17]. Another alternative treatment includes dapsone, which is suitable for individuals who have sulfa allergies but requires monitoring as it can cause hemolytic anemia for individuals with G6PD deficiency [18]. Moreover, atovaquone is an oral suspension that can be used for PCP prophylaxis and is well tolerated [19]. There are many different approaches to PCP prophylaxis and the treatment plan can be altered based on a patient's tolerance, side effects of medication, and their specific case.

## Conclusions

Overall, PCP infection is uncommon without the presence of commonly associated risk factors. As PCP infection is termed an AIDS-defining illness, its occurrence in non-HIV patients is relatively rare. Our patient had a hematological history of MDS-MLD that progressed to AML and was confirmed positive for PCP infection by a positive PCP stain on BAL despite prophylaxis with inhaled pentamidine. As she was a hematopoietic stem cell transplant recipient, PCP was an infection of concern. Although TMP-SMX is typically the primary therapeutic agent and offers broad prophylactic coverage, there is concern regarding myelosuppressive properties and resultant delayed engraftment in the HSCT population. The patient also had a complication in her posttransplant course because of severe acute GVHD, for which she remains on immunosuppressive therapy, which is a risk factor. Considering these factors, the patient was given a regimen of inhaled pentamidine. Despite this, the patient still demonstrated a breakthrough infection, illustrating the need to evaluate different prophylactic strategies in hematopoietic cell transplantation patients such as oral cotrimoxazole, oral dapsone, and oral atovaquone. Additionally, this case highlights the necessity of treating early clinical symptoms in HIV-negative immunocompromised patients for potential PCP infections as mortality rates for these individuals are higher because of delayed diagnosis and clinical symptoms not being obvious for a PCP infection.

## References

[1] Ibrahim A, Chattaraj A, Iqbal Q (2023). Pneumocystis jiroveci pneumonia: a review of management in human immunodeficiency virus (HIV) and non-HIV immunocompromised patients. Avicenna J Med.

[2] Truong J, Ashurst JV (2024). Pneumocystis jirovecii pneumonia. StatPearls.

[3] Kovacs JA, Masur H (2009). Evolving health effects of pneumocystis: one hundred years of progress in diagnosis and treatment. JAMA.

[4] Lee EH, Kim EY, Lee SH (2019). Risk factors and clinical characteristics of Pneumocystis jirovecii pneumonia in lung cancer. Sci Rep.

[5] Gigliotti F, Limper AH, Wright T (2014). Pneumocystis. Cold Spring Harb Perspect Med.

[6] Evans SE, Kottom TJ, Pagano RE, Limper AH (2012). Primary alveolar epithelial cell surface membrane microdomain function is required for Pneumocystis β-glucan-induced inflammatory responses. Innate Immun.

[7] Tasaka S (2015). Pneumocystis pneumonia in human immunodeficiency virus-infected adults and adolescents: current concepts and future directions. Clin Med Insights Circ Respir Pulm Med.

[8] Kanne JP, Yandow DR, Meyer CA (2012). Pneumocystis jiroveci pneumonia: high-resolution CT findings in patients with and without HIV infection. AJR Am J Roentgenol.

[9] Thomas CF Jr, Limper AH (2004). Pneumocystis pneumonia. N Engl J Med.

[10] Noskin GA, Murphy RL, Black JR, Phair JP (1992). Salvage therapy with clindamycin/primaquine for Pneumocystis carinii pneumonia. Clin Infect Dis.

[11] Evernden C, Dowhan M, Dabas R (2020). High incidence of Pneumocystis jirovecii pneumonia in allogeneic hematopoietic cell transplant recipients in the modern era. Cytotherapy.

[12] Roux A, Canet E, Valade S (2014). Pneumocystis jirovecii pneumonia in patients with or without AIDS, France. Emerg Infect Dis.

[13] Gri J, Jain V (2024). Pneumocystis jirovecii pneumonia: a case report. J Med Case Rep.

[14] Li R, Tang Z, Liu F, Yang M (2021). Efficacy and safety of trimethoprim-sulfamethoxazole for the prevention of pneumocystis pneumonia in human immunodeficiency virus-negative immunodeficient patients: a systematic review and meta-analysis. PLoS One.

[15] Haseeb A, Abourehab MA, Almalki WA (2022). Trimethoprim-sulfamethoxazole (Bactrim) dose optimization in Pneumocystis jirovecii pneumonia (PCP) management: a systematic review. Int J Environ Res Public Health.

[16] Robin C, Cordonnier C, Tridello G (2024). Pneumocystis pneumonia after allogeneic hematopoietic cell transplantation: a case-control study on epidemiology and risk factors on behalf of the Infectious Diseases Working Party of the European Society for Blood and Marrow Transplantation. Transplant Cell Ther.

[17] Chiu CY, Ching PR (2023). Incidence of pneumocystis pneumonia in immunocompromised patients without human immunodeficiency virus on intravenous pentamidine prophylaxis: a systematic review and meta-analysis. J Fungi (Basel).

[18] Sangiolo D, Storer B, Nash R (2005). Toxicity and efficacy of daily dapsone as Pneumocystis jiroveci prophylaxis after hematopoietic stem cell transplantation: a case-control study. Biol Blood Marrow Transplant.

[19] Hirai J, Mori N, Kato H, Asai N, Hagihara M, Mikamo H (2023). A case of severe pneumocystis pneumonia in an HIV-negative patient successfully treated with oral atovaquone. Infect Drug Resist.

